# Mr. James Smith Colquhoun

**Published:** 1910-04-30

**Authors:** 


We have to record the death of Mr. James Smith Colquhoun, under tragic circumstances, in Natal. On returning from a professional journey and crossing a swollen river his cart was swept down by the flood and he was drowned. Mr. Colquhoun studied at the Newcastle School and St. Mungo's, Glasgow. He took the L.R.C.P. and L.R.C.S. (Edin.) in 1901, and soon afterwards went to Natal, where he was appointed D.S. of the Underberg District. Later he went to Dannhauser, remaining there as junior partner for two and a half years, and then acquired the whole practice.

